# Genetic structure of Indian populations based on fifteen autosomal microsatellite loci

**DOI:** 10.1186/1471-2156-7-28

**Published:** 2006-05-17

**Authors:** VK Kashyap, Saurav Guha, T Sitalaximi, G Hima Bindu, Seyed E Hasnain, R Trivedi

**Affiliations:** 1 National DNA Analysis Centre, Central Forensic Science Laboratory, 30 Gorachand Road, Kolkata 700014, West Bengal, India; 2 Centre for DNA Fingerprinting and Diagnostics, ECIL Road, Nacharam, Hyderabad 500076, Andhra Pradesh, India; 3 National Institute of Biologicals, A-32, Sector 62, Institutional Area, Noida 201307, Uttar Pradesh, India

## Abstract

**Background:**

Indian populations endowed with unparalleled genetic complexity have received a great deal of attention from scientists world over. However, the fundamental question over their ancestry, whether they are all genetically similar or do exhibit differences attributable to ethnicity, language, geography or socio-cultural affiliation is still unresolved. In order to decipher their underlying genetic structure, we undertook a study on 3522 individuals belonging to 54 endogamous Indian populations representing all major ethnic, linguistic and geographic groups and assessed the genetic variation using autosomal microsatellite markers.

**Results:**

The distribution of the most frequent allele was uniform across populations, revealing an underlying genetic similarity. Patterns of allele distribution suggestive of ethnic or geographic propinquity were discernible only in a few of the populations and was not applicable to the entire dataset while a number of the populations exhibited distinct identities evident from the occurrence of unique alleles in them. Genetic substructuring was detected among populations originating from northeastern and southern India reflective of their migrational histories and genetic isolation respectively.

**Conclusion:**

Our analyses based on autosomal microsatellite markers detected no evidence of general clustering of population groups based on ethnic, linguistic, geographic or socio-cultural affiliations. The existence of substructuring in populations from northeastern and southern India has notable implications for population genetic studies and forensic databases where broad grouping of populations based on such affiliations are frequently employed.

## Background

Human diversity in India is defined by 4693 different, documented population groups that include 2205 major communities, 589 segments and 1900 territorial units spread across the country [1]. Anthropologically, the populations are grouped into four major ethnic categories, which include the Australoid, Indo-Caucasoid, Indo-Mongoloid and Negrito populations and linguistically broadly classified as Indo-European, Dravidian, Austro-Asiatic and Sino-Tibetan speakers. The complex structure of the Indian population is attributed to incessant, historical waves of migrations into India, the earliest, by the Austric speakers around 70,000 years ago, followed by the Dravidian speakers from middle-east Asia and the Sino-Tibetan speakers from China and southeast Asia around 8000 to 10,000 years ago. The last major migration is believed to have occurred around 4000 years ago by several waves of Indo-European speakers [2]. Earlier genetic studies to understand the prevailing diversity among extant Indian populations analyzing populations that were predefined either based on ethnicity, language, culture or geography have interpreted existence of different levels of genetic relationships among population groups [3-6] that broadly attest the theories of migration and assimilation of different populations. However, recent molecular analyses have also asserted genetic similarity across populations spread over diverse geographic regions of the country, revealing a gradation of genetic lineages underscoring the genetic correlation amongst populations [7,8].

The striking social attribute of the Indian populations is their strict practice of endogamy across all social ranks that has resulted in emergence of diverse population-specific social traditions and formation of distinct linguistic dialects due to subsequent isolation of populations. Although uniparental, biallelic markers have deciphered the common major Paleolithic contributions [9], resolution of many sub-lineages is still awaited in order to decipher finer genetic signatures defining populations that have resisted admixture for centuries. Patterns of variation across recently diverged populations can be successfully characterized with fast-evolving microsatellite markers [10][11][12]. Genetic drift among isolated, small populations manifests as characteristic allele frequency patterns that have been recently effectively characterized to identify genetic clusters that corresponded well with predefined geographically or linguistically similar populations [13].

With these rationales, we have analyzed 15 highly polymorphic autosomal microsatellite markers including 13 core forensic loci, which have been extensively used to reveal the ethnological and anthropological affinity of diverse populations ([10][11][12], [14][15][16][17][18][19]). In order to decipher if geographic proximity,  linguistic, ethnic and socio-cultural affiliations  have played a role in genetic differentiation of  extant Indian populations these markers were analyzed  in over 3522 individuals drawn from 54 endogamous  populations representing major ethnic and linguistic  groups spread across diverse geographic regions of the  country (Table 1). Distribution of alleles across populations was evaluated to ascertain presence of group-specific patterns if any. Extent of molecular variance evident among pre-defined groups based on ethnicity, language, geography and socio-cultural hierarchy was evaluated to determine if such classifications were supported genetically. In addition, a model-based clustering algorithm was applied to infer population groups differentiated by their characteristic allele frequencies and to detect presence of cryptic population subdivisions.

## Results

**Figure 1:**
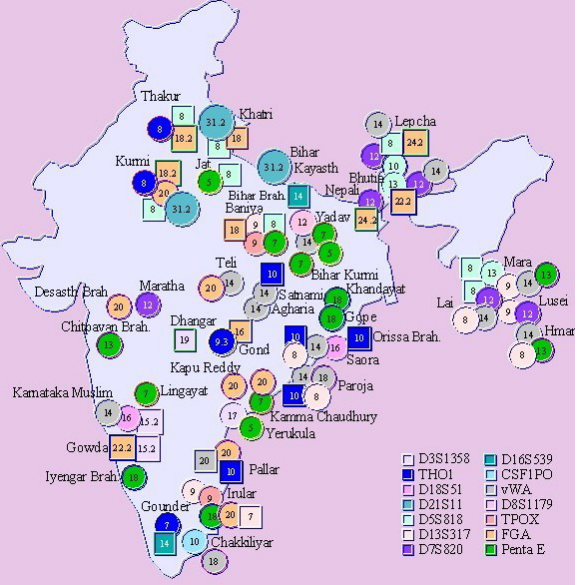
Alleles with significant distribution among the different groups of India for the studied microsatellite markers. ○ represents alleles occurring at a high frequency and □ denotes unique alleles present in a population.

A number of alleles of the different microsatellite loci analyzed were found to be present unique to specific populations with discernable distribution along geographic and ethnic affiliations evident only among few of the populations. Populations like the Gond (a tribal population) from Chattisgarh; Irular, Chakkiliyar, Gounder and Pallar (Australoid populations) from the southern state of Tamil Nadu; showed genetic isolation, evident from the presence of alleles confined within these populations (Figure 1). On the contrary, allele 15.2 of the D3S1358 locus was found to be prevalent among the Gowda and Muslims in the state of Karnataka and allele 18.2 of the FGA locus was present among the Thakur and Kurmi of Uttar Pradesh exhibiting a regional distribution. Sharing of allele 24.2 of the FGA locus was also observed between Lepcha and the Nepali of Sikkim, who share similar ethnic and geographic origins.

Significantly, most frequent alleles were shared among some ethnically and linguistically related populations. The populations of Sikkim, Lai and Lusei of Mizoram that shared Mongol ancestry had a high frequency of allele 12 of the D7S820 locus. Analogous results were obtained for allele 13 of the D5S818 locus, which was in high incidence amongst the Bhutia of Sikkim and Mara of Mizoram. The Indo-Caucasoids, Lingayat of Karnataka; Yadav and Baniya of Bihar, and the geographically proximate Australoid, Kurmi had allele 7 of the Penta E locus in high frequency. Allele 18 of the same locus was present in high frequencies among the Dravidian speaking Australoids, Gowda of Karnataka; Irular of Tamil Nadu as well as among the Indo-European speaking Indo-Caucasoids, Khandayat and Gope of Orissa.

Analysis of molecular variance (Table 2) failed to support the geographic, ethnic, linguistic or socio-cultural grouping of Indian populations suggesting little variation between the different groups. We then employed a cluster-based algorithm to ascertain the extent to which the observed discrete patterns of allele distributions would delineate populations. In order to maintain uniformity of estimated probabilities across runs for a given value of K with large datasets [20], we initially used small K to analyze the 54 populations in this study and then subdivided the dataset into smaller groups to dissect the regional diversity.

**Figure 2:**
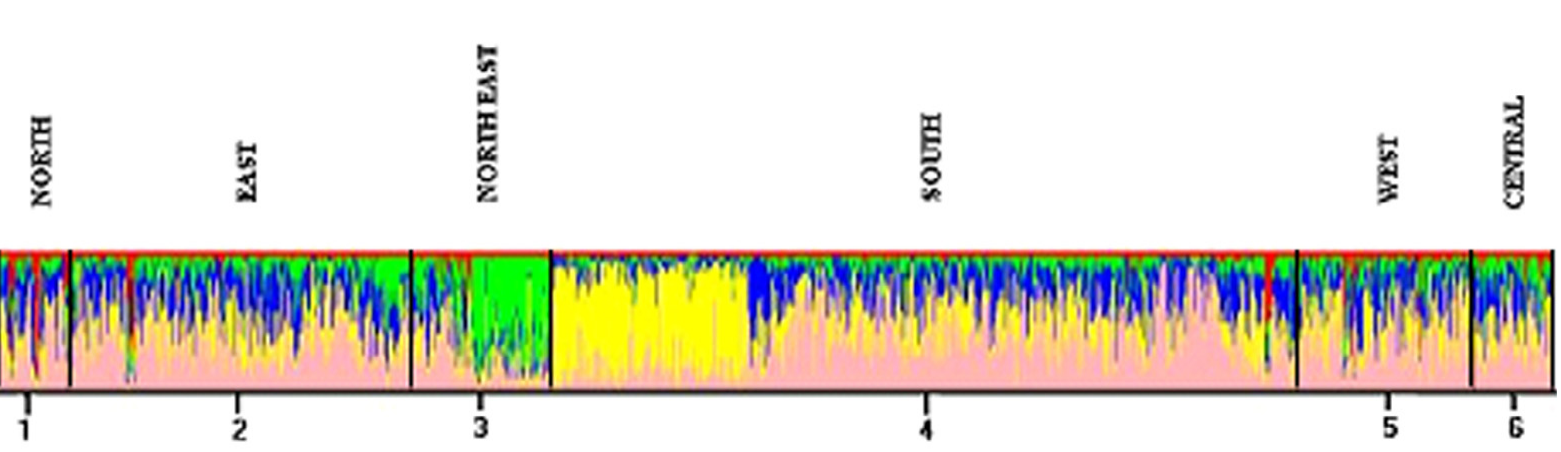
Bar plot of estimation of the membership coefficient (Q) for each individual of the Indian population grouped on geographic distribution. Each individual is represented by a thin vertical line, which is partitioned into *K *colored segments that represent the individual's estimated membership fractions in *K *clusters. Black lines separate individuals of different population groups based on geography. Population groups are labeled below the figure, with their geographical affiliations above it. The figure shown for *K *= 5 is based on the highest probability run at that *K*.

In the countrywide dataset, at K = 5, associated with maximum posterior probability (Table 3), individuals displayed partial membership to multiple clusters with some populations exhibiting distinctive identities that did not correspond to geographic, linguistic or ethnic affiliation (Figure 2). Populations such as Thakur and Khatri from Uttar Pradesh and Baniya from Bihar showed similarity with southern populations such as Naikpod Gond and Chenchu from Andhra Pradesh and with a few individuals from Maharashtra and Lepcha of Sikkim. Populations from the northeastern state of Mizoram exhibited a distinct clustering, different from populations of similar ethnicity from Sikkim, while some individuals from Saora and Gope from the eastern state of Orissa shared a similar degree of membership as the Mizoram populations. Of the southern populations, those from Karnataka and Andhra Pradesh were differentiated into two groups with populations from Tamil Nadu exhibiting split membership to both groups.

**Figure 3:**
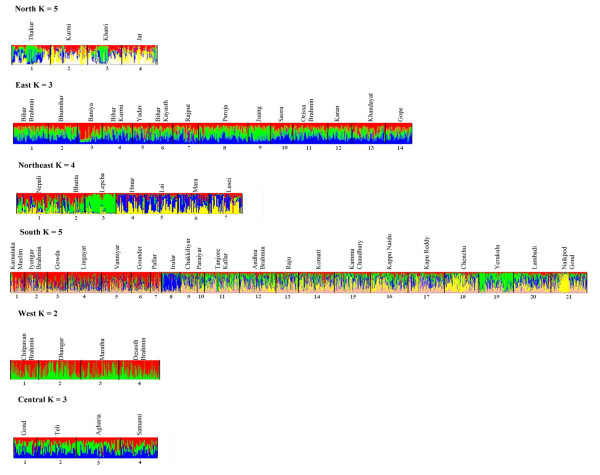
Estimated population structure in different geographic regions. Bar plot estimation figures for North, East, Northeast, South, West, and Central were based on the highest probability run at that K.

At the regional level (Figure 3), amongst northern Indian populations, at K = 5, where the highest posterior probability was associated, Thakur were identified to be distinct from Jat and Uttar Pradesh Kurmi. The Khatri were found substructured with few individuals exhibiting membership similar to the Thakur.

In the East, Bihar Brahmin, Bhumihar, Kayasth, Rajput, Yadav, Bihar Kurmi, Orissa Brahmin, Khandayat, Karan, Juang and Paroja shared similar membership to multiple clusters revealing a common genetic structure. Baniya of Bihar were found similar to two of the northern Indian populations while Gope and few individuals from Saora of Orissa shared similar identities as the populations from Mizoram of northeastern India.

The northeastern populations from Mizoram were identified to be distinct from those of Sikkim. Three clusters were evident with Hmar, Mara, Lai and Lusei of Mizoram all representing one group while Lepcha of Sikkim were distinct representing the second group and the third group comprised Nepali and Bhutia of Sikkim.

In the south, Lingayat, Gowda, Brahmin and Muslim of Karnataka along with Vanniyar, Gounder and Pallar of Tamil Nadu separated from rest of the populations. Irular of Tamil Nadu and Yerukula of Andhra Pradesh presented distinct identities while Chenchu and Naikpod Gond of Andhra Pradesh exhibited similar affinities. Rest of the populations from Tamil Nadu; Chakkiliyar, Paraiyar, Tanjore Kallar and from Andhra Pradesh; Brahmin, Raju, Komati, Kamma Chaudhury, Kapu Naidu, Reddy and Lambadi displayed mixed membership to multiple clusters.

Populations from western and central India showed absence of any distinct grouping with individuals having symmetrical membership across inferred clusters. The above results reveal genetic similarity across populations with a few presenting distinct identities that did not follow traditional groupings of geography, language or ethnicity. Populations from southern India and northeastern India largely exhibited structuring while most Indian populations shared similar membership in multiple clusters.

## Discussion

Contemporary molecular studies on Indian populations were focused to uncover the genetic relationship among geographically, linguistically or ethnically related populations [21-25]. Recently, few studies involving a larger number of populations have correlated the genetic relatedness of the populations with linguistic [6] or socio-cultural affinities, [3,5] though genetic uniformity across populations has also been largely observed [7,8]. The current study employs microsatellite markers to decipher allele frequency changes that would effectively detect recently isolated populations whose times of divergences were shorter than those detectable by uniparental markers. Distribution of alleles across the microsatellite loci studied among the populations predominantly demonstrates the occurrence of alleles unique only to a few populations (Figure 1). This pattern is probably due to the result of genetic isolation and drift experienced by the populations that follow strict endogamous practices. The distribution of the most frequent allele was in general, uniform across populations suggesting their common origin. Earlier reports have also suggested geographic contiguity favoring gene flow among populations [26]. Although ethnic and geographic propinquity were discernible from the allele distribution patterns across few populations in the current study, no consistent pattern across all populations of any particular group was observed. This was also evident from the analysis of molecular variance that failed to support any grouping; ethnic, linguistic, geographic or socio-cultural in contributing to the extant genetic structure of Indian populations.

The immense diversity within the ethnic and linguistic affiliations of the populations inhabiting India had always been a debatable issue, whether some of them had originated indigenously or were the results of earlier migrations [27-29]. The distinct grouping of the populations of Mizoram (Figure 3) does concord with earlier reports [4,30] that northeastern India was peopled by migration of Tibeto-Burman speakers from East Asia. However, Tibeto-Burman speaking populations of Sikkim grouped separately and exhibited considerable gene-flow with non-Tibeto-Burman speakers. It is probable that these two regions were peopled by different waves of migration from Southeast and East Asia. Interestingly, eastern Indian populations; Saora and Gope also exhibit similarities to the populations of Mizoram indicating shared genetic ancestry. Though the Lepcha were distinct at the highest-likelihood run for K = 4 (Figure 3), in other runs with lower K, they grouped with the rest of the populations from Nepal (data not shown).

Majority of the Indian populations in general exhibited extensive admixture with each population displaying membership to multiple clusters. Populations such as Khatri, Baniya, Chenchu, Yerukula and Naikpod Gond, however, were substructured. Interestingly, populations comprising the southern Indian region exhibited substructuring with a number of populations clustering into a separate group while the rest were found similar to the general Indian population structure. This group comprising Iyenger Brahmin, Lingayat, Gowda and Muslim from Karnataka and Gounder, Vanniyar and Pallar from Tamil Nadu probably represents those populations that have resisted recent geneflow, and accumulated characteristic allele frequencies because of genetic drift leading to their differentiation from the rest of the populations. In addition, Irular of Tamil Nadu and Yerukula of Andhra Pradesh were found distinctive while Chenchu and Naikpod Gond of Andhra Pradesh grouped together. However, these populations at lower K grouped into clusters similar to those of Tanjore Kallar, Paraiyar and Chakkiliyar of Tamil Nadu and Brahmin, Raju, Komati, Kamma Chaudhury, Kappu Naidu, Kapu Reddy and Lambadi of Andhra Pradesh.

## Conclusion

Our analyses failed to reveal any genetic groups that correlate to language, geography, ethnicity or socio-cultural affiliation of populations. Of course, the absence of evidence of structuring of the Indian populations based on ethnic, linguistic, geographic or socio-cultural affiliations may be related to the ascertainment bias of selection of these highly polymorphic forensic microsatellite markers. Future studies employing a large number of microsatellites/SNPs might yield higher resolution to decipher stronger associations between populations. The occurrence of few populations distinct from the general populace suggests genetic drift due to isolation of such populations have resulted in their characteristic allele frequencies. This cryptic population structure would have significant implications in forensic investigations where computations of statistical significance of a DNA match rely on ethnic identities often defined by the country of origin. The existence of substructuring in populations from northeastern and southern India also cautions against broad grouping of populations based on geographic, ethnic or linguistic affiliation that are frequently employed in population genetic studies.

## Methods

A total of 3522 consenting individuals from fifty four populations belonging to three major ethnic groups and affiliated to four major language families from across the country were included in this study (Table 1) after approval of the ethical committee of the Central Forensic Science Laboratory. To ensure representations from all groups, information on geographic origin, ethnicity and linguistic affiliation were recorded for every individual sampled.

DNA was extracted either from blood or buccal swabs by standard methods [31]. Amplification was carried out using the Power Plex^®^16 system (Promega Corporation, Madison, USA) or AmpF*l*STR ^®^Identifiler™ PCR Amplification Kit (Applied Biosystems, Foster City, California, USA) that coamplify fifteen microsatellite loci according to manufacturers' specifications. The amplified products were separated on a denaturing 5% polyacrylamide gel using the ABI Prism™ 377 DNA Sequencer (PE Applied Biosystems, Foster City, CA, USA). The genotypes were analyzed with GeneScan^® ^Analysis 3.1, Genotyper^® ^2.5 (PE Applied Biosystems, Foster City, CA, USA) and PowerTyper™ 16 Macro v2 (Promega Corporation, Madison, USA) softwares.

Analysis of molecular variance, AMOVA [32], was performed by Arlequin 2.0 software using all 15 loci to ascertain which of the attributes; ethnicity, social hierarchy, geographic or linguistic affiliation of the Indian populations contribute maximum to the extant genetic structure. Significance of the AMOVA values was estimated by use of 10,000 permutations.

We used a model-based clustering method for inferring population groups using genotype data consisting of unlinked markers as implemented in *Structure *2.1 program [33]. The model assumes there are *K *populations (where *K *may be unknown), each of which is characterized by a set of allele frequencies at each locus. Individuals in the sample are assigned probabilistically to populations, or jointly to two or more populations if their genotypes indicate they are admixed. Each run used 100,000 estimation iterations for K = 2 to 8 after a 20,000 burn-in length. Each run was carried out several times to ensure consistency of the results. Posterior probabilities for each K were computed for each set of runs.

## Competing interests

The author(s) declare that they have no competing interests.

## Authors' contributions

VKK designed the course of the study and contributed significantly in manuscript preparation. SG carried out statistical analysis and participated in manuscript preparation. TS analyzed the data and drafted the manuscript. GHB performed experiments on Andhra Pradesh samples and participated in manuscript preparation. SEH provided analytical inputs for manuscript preparation. RT provided critical information for data processing and manuscript preparation.

All authors read and approved the final manuscript.

**Table 1:** Ethnic, linguistic and geographical affiliations of Indian populations included in the study

	Population	Sample size	Ethnicity	Language family	Area of sampling	Microsatellite data source
1	Jat	48	Caucasoid	Indo-European	Uttar Pradesh	[34]
2	Khatri	47	Caucasoid	Indo-European	Uttar Pradesh	[34]
3	Kurmi	52	Australoid	Austro-Asiatic	Uttar Pradesh	[34]
4	Thakur	52	Caucasoid	Indo-European	Uttar Pradesh	[34]
5	Desasth Brahmin	107	Caucasoid	Indo-European	Maharashtra	[35]
6	Dhangar	112	Australoid	Indo-European	Maharashtra	[35]
7	Chitpavan Brahmin	76	Caucasoid	Indo-European	Maharashtra	[35]
8	Maratha	102	Caucasoid	Indo-European	Maharashtra	[35]
9	Agharia	53	Caucasoid	Indo-European	Chattisgarh	[36]
10	Gond	28	Australoid	Dravidian	Chattisgarh	[36]
11	Satnami	44	Caucasoid	Indo-European	Chattisgarh	[36]
12	Teli	47	Caucasoid	Indo-European	Chattisgarh	[36]
13	Baniya	45	Caucasoid	Indo-European	Bihar	[37]
14	Bhumihar	65	Caucasoid	Indo-European	Bihar	[38]
15	Bihar Brahmin	58	Caucasoid	Indo-European	Bihar	[38]
16	Bihar Kayasth	45	Caucasoid	Indo-European	Bihar	[38]
17	Bihar Kurmi	49	Australoid	Austro-Asiatic	Bihar	[37]
18	Rajput	58	Caucasoid	Indo-European	Bihar	[38]
19	Yadav	40	Caucasoid	Indo-European	Bihar	[37]
20	Bhutia	32	Mongoloid	Tibeto-Burman	Sikkim	[39]
21	Lepcha	44	Mongoloid	Tibeto-Burman	Sikkim	[39]
22	Nepali	63	Mongoloid	Tibeto-Burman	Sikkim	[39]
23	Hmar	40	Mongoloid	Tibeto-Burman	Mizoram	[40]
24	Lai	46	Mongoloid	Tibeto-Burman	Mizoram	[40]
25	Lusei	46	Mongoloid	Tibeto-Burman	Mizoram	[40]
26	Mara	44	Mongoloid	Tibeto-Burman	Mizoram	[40]
27	Gope	60	Caucasoid	Indo-European	Orissa	[41]
28	Juang	50	Australoid	Austro-Asiatic	Orissa	[42]
29	Karan	62	Caucasoid	Indo-European	Orissa	[41]
30	Khandayat	62	Caucasoid	Indo-European	Orissa	[41]
31	Orissa Brahmin	57	Caucasoid	Indo-European	Orissa	[41]
32	Paroja	77	Australoid	Dravidian	Orissa	[42]
33	Saora	35	Australoid	Austro-Asiatic	Orissa	[42]
34	Gowda	59	Australoid	Dravidian	Karnataka	[43]
35	Iyengar Brahmin	65	Caucasoid	Dravidian	Karnataka	[43]
36	Karnataka Muslim	45	Caucasoid	Dravidian	Karnataka	[43]
37	Lingayat	98	Caucasoid	Dravidian	Karnataka	[43]
38	Chakkiliyar	49	Australoid	Dravidian	Tamil Nadu	[44]
39	Gounder	56	Australoid	Dravidian	Tamil Nadu	[44]
40	Irular	54	Australoid	Dravidian	Tamil Nadu	[44]
41	Pallar	33	Australoid	Dravidian	Tamil Nadu	[45]
42	Tanjore Kallar	101	Australoid	Dravidian	Tamil Nadu	[45]
43	Vanniyar	86	Australoid	Dravidian	Tamil Nadu	[45]
44	Paraiyar	21	Australoid	Dravidian	Tamil Nadu	[45]
45	Andhra Brahmin	106	Caucasoid	Dravidian	Andhra Pradesh	[46]
46	Raju	66	Australoid	Dravidian	Andhra Pradesh	[46]
47	Komati	104	Australoid	Dravidian	Andhra Pradesh	[46]
48	Kamma Chaudhury	106	Australoid	Dravidian	Andhra Pradesh	[47]
49	Kappu Naidu	107	Australoid	Dravidian	Andhra Pradesh	[47]
50	Kapu Reddy	107	Australoid	Dravidian	Andhra Pradesh	[47]
51	Chenchu	100	Australoid	Dravidian	Andhra Pradesh	[48]
52	Yerukula	101	Australoid	Dravidian	Andhra Pradesh	[48]
53	Naikpod Gond	104	Australoid	Dravidian	Andhra Pradesh	[48]
54	Lambadi	108	Caucasoid	Indo-European	Andhra Pradesh	[48]

**Table 2:** Analysis of molecular variance across different groups of Indian populations

			Percentage of Variation			
				
Sample	Number of groups	Number of populations	Within population	Among populations within groups	Among groups	F_st_
India	1	54	98.16	1.84	-	0.01840
Geography	6	54	97.95	1.26	0.79	0.02051
Language	3	54	97.97	1.51	0.51	0.02025
Ethnicity	4	54	97.99	1.59	0.43	0.02012
Caste & Tribe	2	54	98.05	1.76	0.19	0.01953
Caste – Geography	6	39	98.48	1.00	0.53	0.01523
Tribe – Geography	5	15	96.74	1.93	1.32	0.03258

**Table 3:** Estimates of log probability of data under admixture model for geographic groups of Indian populations

Run	K	ln pr(x/k)	p(k/x)
1	2	-190467.3	0
2	3	-190000.7	0
3	4	-189591	6.95 × 10^-15^
4	5	-189558.4	0.99
5	6	-189697.2	5.24 × 10^-61^
6	7	-189576.2	1.86 × 10^-8^
7	8	-189822.7	0
